# Eprinomectin pour-on (EPRINEX® Pour-on, Merial): efficacy against gastrointestinal and pulmonary nematodes and pharmacokinetics in sheep

**DOI:** 10.1186/s12917-017-1075-7

**Published:** 2017-05-30

**Authors:** Dietmar Hamel, Antonio Bosco, Laura Rinaldi, Giuseppe Cringoli, Karl-Heinz Kaulfuß, Michael Kellermann, James Fischer, Hailun Wang, Katrin Kley, Sandra Mayr, Renate Rauh, Martin Visser, Thea Wiefel, Becky Fankhauser, Steffen Rehbein

**Affiliations:** 1 Merial GmbH, Kathrinenhof Research Center, Walchenseestr. 8-12, 83101 Rohrdorf, Germany; 2 Department of Veterinary Medicine and Animal Production, University of Naples Federico II, Via della Veterinaria, 1, 80137 Naples, Italy; 3 Tierarztpraxis Hoffmann, Untere Schulstraße 8, 38875 Elbingerode, Germany; 4 Merial, Inc., North Brunswick Research Center, 631 Route 1 South, North Brunswick, NJ 08902 USA; 5 Merial, Inc., 3239 Satellite Blvd., Duluth, GA 30096-4640 USA

**Keywords:** Eprinomectin, Topical, Gastrointestinal nematodes, Lungworms, Pharmacokinetics, Sheep

## Abstract

**Background:**

The anthelmintic efficacy of the 0.5% *w*/*v* topical formulation of eprinomectin (EPN), EPRINEX® Pour-on (Merial) when administered at 1 mg/kg body weight was evaluated in sheep in two dose confirmation laboratory studies and one multicenter field study. In addition, the pharmacokinetics of EPN when administered at that dosage to adult sheep was determined.

**Results:**

In the two dose confirmation studies, which included 10 sheep each, sheep treated with topical EPN had significantly (*p* < 0.05) fewer of the following nematodes than the untreated sheep with overall reduction of nematode counts by >99%: adult *Dictyocaulus filaria*, *Haemonchus contortus*, *Teladorsagia circumcincta*(*pinnata/trifurcata*), *Trichostrongylus axei*, *T. colubriformis*, *T. vitrinus*, *Cooperia curticei*, *Nematodirus battus*, *Strongyloides papillosus*, *Chabertia ovina* and *Oesophagostomum venulosum*, and inhibited fourth-stage *Teladorsagia* larvae.

A total of 196 sheep harboring naturally acquired gastrointestinal nematode infections were included in the field efficacy study at two sites each in Germany (48 Merino x Ile de France lambs, 52 adult Merino females) and in Italy (adult male and female Bagnolese, Lacaune, Lacaune x Bagnolese, Bagnolese x Sarda sheep; 48 animals per site). Animals were blocked on pre-treatment body weight and within each block, one animal was randomly assigned to the control (untreated) group and three animals were randomly assigned to be treated with topical EPN. Examination of feces 14 days after treatment demonstrated that, relative to the controls, topical EPN-treated sheep had significantly (*p* < 0.0001) lower strongylid egg counts. Reduction was ≥97% at each site and 98.6% across all sites.

Pharmacokinetics of EPN following single treatment with topical EPN were determined in eight ~4.5 year old female Merino cross sheep based on the analysis of plasma samples which were collected from two hours to 21 days following treatment. The main pharmacokinetic parameters were: C_max_ 6.20 ± 1.71 ng/mL, AUC_last_ 48.8 ± 19.2 day*ng/mL, T_max_ 3.13 ± 2.99 days and T_1/2_ 6.40 ± 2.95 days.

No treatment-related health problems or adverse drug events were observed in any study.

**Conclusion:**

These studies demonstrated 0.5% *w*/*v* EPN administered topically at 1 mg/kg body weight to be highly efficacious against a broad range of ovine gastrointestinal nematodes and *D. filaria* lungworms and well tolerated by sheep of different ages, breeds, gender and physiological status.

## Background

Because of their ubiquitous occurrence, nematode endoparasites are a major concern to sheep farmers and are an important drain of resources worldwide. Nematode parasitism negatively impacts the production (meat, milk, wool) and reproduction of sheep and has the capability to seriously compromise the health and welfare of the animals. Even subclinical nematode infections cause losses of productivity as demonstrated repeatedly by treatment-induced improved performance e. g., [1–10]. Therefore, a prerequisite for economically sustainable sheep farming and efficient production is the effective control of ovine nematode parasites [8, 11].

Eprinomectin is a macrocyclic lactone registered as a broad spectrum endectocide as a 0.5% *w*/*v* topical formulation (EPRINEX® Pour-on, Merial) for use in cattle. In this formulation, eprinomectin dosed at 0.5 mg per kg body weight is characterized by a broad safety margin and a zero milk withholding in dairy cows due to a low milk partitioning coefficient, an exceptional pharmacokinetic property within the macrocyclic lactone class of anthelmintics [12, 13]. The excellent endoparasiticidal efficacy of eprinomectin in sheep has been known for more than 20 years because experimentally infected sheep dosed orally were used for screening avermectin/milbemycin analogs in the effort to identify a candidate compound allowing the use in all classes of cattle, including lactating animals [12]. However, reports on the topical treatment of sheep with eprinomectin have been published only quite recently [14–20].

While there are drugs from all anthelmintic classes available for effective treatment of ovine endoparasites, most products are not authorized for use in lactating dairy animals or require a period of withholding the milk because of the levels of residues excreted with milk. Products without disclaimer against use in lactating dairy sheep are of particular importance for the commercial sheep farming in the Mediterranean region where about two thirds of the world’s sheep milk is produced [21].

Based on studies determining the excretion of eprinomectin in the milk of lactating sheep (Merial, unpublished data), 0.5% *w*/*v* eprinomectin (EPRINEX® Pour-on, Merial) administered at 1 mL per kg body weight (equivalent to 1 mg eprinomectin per kg body weight) topically to lactating sheep has been recently granted zero hours milk withdrawal by the European Medicines Agency.

Here we present the results of a series of four studies (two dose confirmation laboratory studies, one multicenter field efficacy study and one pharmacokinetic study) which were conducted between 2013 and 2015 in order to support the market authorization in sheep of 0.5% *w*/*v* topical formulation of eprinomectin (EPRINEX® Pour-on, Merial) when administered at 1 mg per kg body weight.

## Methods

This series of studies consisted of two dose confirmation laboratory studies (Studies 1 and 2), one multicenter field efficacy study (Study 3), and one pharmacokinetic study (Study 4). The design of the Studies 1, 2 and 3 was in accordance with the International Cooperation on Harmonisation of Technical Requirements for Registration of Veterinary Medicinal Products (VICH) GL7, “Efficacy of Anthelmintics: General Requirements” and GL13, “Efficacy of Anthelmintics: Specific Recommendations for Ovine” [22] and the “World Association for the Advancement of Veterinary Parasitology (W.A.A.V.P.) second edition of guidelines for evaluating the efficacy of anthelmintics in ruminants (bovine, ovine, caprine)” [23]. The studies were conducted in compliance with VICH GL9, entitled Good Clinical Practice and were performed as blinded studies, i.e., all personnel involved in collecting efficacy data and making health observations were masked as to the treatment assignment of the animals.

Study 4 was conducted in accordance to Guidelines for the Conduct of Pharmacokinetic Studies in Target Animal Species, EMEA/CVMP/133/99-FINAL.

### General study design

Studies 1, 2 and 3 were conducted as randomized block design studies with blocks of two (Studies 1 and 2) or four (Study 3) animals formed based on pre-treatment body weight. Within blocks, animals were allocated at random to treatment groups, Control (untreated) or to be treated with 0.5% *w*/*v* eprinomectin (EPRINEX® Pour-on, Merial) at 1 mL per 5 kg of body weight topically (1 mg eprinomectin per kg body weight). As per VICH GL 7, control (untreated) to 0.5% *w*/*v* eprinomectin (treated) ratio was 1:1 in the dose confirmation studies (Studies 1 and 2); however, the ration was 1:3 in the multicenter field efficacy study (Study 3) in order to gain further experience on the test product in a larger number of animals of different breeds, age, body weight, gender and physiological status. All eight sheep enrolled in Study 4 were treated with 0.5% *w*/*v* eprinomectin at 1 mL per 5 kg of body weight topically.

Pre-treatment body weight obtained with verified scales on Day −5 (Study 3/Sites 3 and 4), or Day −1 (Studies 1, 2, 3/Sites 1 and 2, and Study 4) was used for allocation and dose calculation, as appropriate. The calculated dose was rounded up to the next 0.5 mL (Study 4) or 1.0 mL (Studies 1, 2 and 3) increment, if it was not an exact 0.5 mL or 1.0 mL increment, respectively.

Treatment was administered once at Day 0 topically along the back line, from the withers to the tail head using appropriately sized syringes. For administration of formulation, the fleece was parted, and the formulation was administered directly onto the skin of the sheep.

In each study, general health observations were carried out daily. In addition, animals were observed hourly for the first four hours after treatment for reactions to treatment.

### Study Animals: Studies 1, 2, 3 and 4

Sheep of different breeds, age, body weight, gender and physiological status were included in the four studies (Table 1). While the animals used in Study 3 were owned by private sheep farmers, sheep utilized in Studies 1, 2 and 4 were bought from commercial farms. None of the animals were treated with macrocyclic lactone products within six weeks of the start of the study.

**Table 1:** Description of study animals

Study	Number of animals	Breed	Sex	Age (range)	Pre-treatment (Days −5 to −1) body weight (kg), range)
1	20	Merino	Male	~5–6 months	33.2–46.0
2	20	Merino Cross	Female^a^	~3–6 years	37.4–76.2
3, Site 1, Germany 1	48	Merino x Ile de France	Female	~6 months	25.2–44.2
3, Site 2, Germany 2	52	Merino	Female^a^	~2–7 years	37.8–81.6
3, Site 3, Italy 1	48	Bagnolese (44), Lacaune x Bagnolese (4)	Male (3), female (45)^b^	~1–6 years	55.5–104.7
3, Site 4, Italy 2	48	Bagnolese (26), Lacaune (16), Sarda x Bagnolese (5), Lacaune x Bagnolese (1)	Female^c^	~2–6 years	40.2–71.4
4	8	Merino Cross	Female^a^	~4.5 years	66.8–101.8

^a^Dry, not pregnant

^b^Dry, not pregnant (15); dry, pregnant (9); lactating, not pregnant (21)

^c^Lactating, not pregnant

Sheep included in Studies 1, 2 and 4 were kept indoors on straw, and following allocation to treatment groups, animals were housed in individual pens to prevent them from having physical contact with others. Animals were offered a roughage-based diet for ad libitum consumption. Sheep included at the four sites in the multicenter field Study 3 (Sites 1 and 2, Germany; Sites 3 and 4, Italy) were grazed on permanent pastures with the study animals (treated and untreated sheep) grazing together with sheep not enrolled in the study (remaining sheep at sites). Animals in all studies had continuous access to water.

Animals in Study 1 were tested negative for patent gastrointestinal and pulmonary nematode infections prior to first inoculation with gastrointestinal nematodes and *Dictyocaulus filaria* lungworms. At commencement of Studies 2 and 3, all sheep harbored naturally acquired gastrointestinal nematode infections as demonstrated through shedding strongylid (other than *Nematodirus*) eggs prior to treatment; in addition, *Nematodirus* eggs, *Trichuris* eggs, *Moniezia* eggs and/or protostrongylid larvae were recovered from the feces of various animals.

### Fecal Examination: Studies 1, 2 and 3

In Study 1, rectal fecal samples were collected from all animals and examined to confirm the absence of patent gastrointestinal and pulmonary nematode infections seven days prior to the initiation of experimental nematode infections. In Studies 2 and 3, rectal fecal samples were collected from all animals ten or five days prior to treatment, respectively, and examined to confirm the presence of natural infection of the animals with gastrointestinal nematodes and/or lungworms. In order to estimate the efficacy of the treatment in terms of the reduction of fecal nematode egg counts in Study 3, individual fecal samples were collected in addition 14 days after treatment and examined.

For fecal egg counting a modified McMaster method with one egg counted representing 10 eggs per gram of feces (EPG) was used with saturated sodium chloride solution for floatation [24] in Studies 1, 2 and 3/Sites 1 and 2. Samples collected in Study 3/Sites 3 and 4 were examined using the FLOTAC dual technique (sensitivity = 6 EPG) [25]. For lungworm larval recovery, 10-g (Studies 1, 2 and 3/Sites 1 and 2) or 5-g (Study 3/Sites 3 and 4) fecal samples were subjected to the Baermann technique [24] to establish lungworm larval counts per gram of feces. When present, eggs were referred to as ‘strongylid’ (nematode genera including *Bunostomum, Chabertia, Cooperia, Haemonchus, Oesophagostomum*, *Teladorsagia,* and *Trichostrongylus*), *Nematodirus* (a strongylid which was identified and counted independently), *Strongyloides* and/or *Trichuris*. Other findings in the fecal examination (*Moniezia* eggs and protostrongylid lungworm larvae) were recorded.

In addition, fecal culture procedures were employed for the identification of the larvae of strongylid nematodes developing from the eggs excreted by the sheep in the multicenter field Study 3. Composite fecal cultures were performed utilizing the fecal samples subjected to lungworm larval recovery (fecal samples of all animals combined prior to treatment; fecal samples of animals combined by treatment group post-treatment) to determine composition by genera. For coproculture, samples of fecal composites were mixed with vermiculite and incubated for seven days after which the third-stage larvae were harvested. Per culture, 100 larvae were identified to genus using standard morphological identification keys [24, 26].

### Inoculation of Sheep: Study 1

Sheep of Study 1 were inoculated with infective third-stage larvae (L3) of gastrointestinal and pulmonary nematode species by oral gavage. The inoculation schedule was designed so that nematodes were expected to be adults on Day 0 (= day of treatment): Day −56, *Chabertia ovina*, ~800 L3 per animal; Day −35, *D. filaria*, ~500 L3 per animal and *Oesophagostomum venulosum*, ~800 L3 per animal; Day −28, *Teladorsagia circumcincta*(*pinnata*/*trifurcata*), ~8000 L3 per animal; Day −25, *Haemonchus contortus*, ~2000 L3 per animal; Day −23, *Trichostrongylus axei*, ~5000 L3 and *Nematodirus battus*, ~2000 L3 per animal; Day −21, *T. colubriformis* and *Cooperia curticei*, ~5000 L3 per species and animal. The parasites used were recent field isolates from Germany as defined per VICH GL 7 [22]. The number of larvae given was generally in accord with the W.A.A.V.P. guidelines for testing of anthelmintics in ruminants [23].

### Parasite counts: Studies 1, 2 and 3

In Studies 1 and 2, all animals were humanely euthanized and organs (the lungs, abomasum, small intestine and large intestine including cecum) were collected for parasite recovery and count 14 days after treatment administration. In Study 3, two sentinel animals with the same history as and thus representative of the study animals were randomly selected at each of the sites and necropsied prior to treatment of the study animals for parasite recovery and count.

Lungs were examined completely for lungworms by lengthwise opening of all accessible air passages. The contents of the abomasum, small and large intestines were collected separately and diluted with water. Abomasum and small intestine were incubated (saline soak) overnight to recover mucosal stages of the parasites for identification and counting. To facilitate isolation and counting of nematodes, organ contents and soaks were screened over sieves of appropriate mesh sizes (abomasum and small intestine contents: 150 μm; large intestine content: 250 μm; abomasal soak: 40 μm) to remove the debris. Gastrointestinal nematode counts were made on 10% aliquots (abomasum, abomasum soak and small intestine; Studies 1, 2 and 3), 20% aliquots (large intestine; Study 1) or total content (large intestine; Studies 2 and 3); cestodes were collected directly from the small intestines during processing and counted totally. Counts of each nematode species for each animal were calculated by multiplying the number of worms actually counted from each organ by the aliquot factor and summing over all organs.

*Teladorsagia* male nematodes were identified to ‘morphs’ (*T*. *circumcincta*, *pinnata* and *trifurcata*), based on their distinct morphological characters. However, in accepting the concept of polymorphism [27, 28], total worm count was presented as ‘*T*. *circumcincta*(*pinnata/trifurcata*)’ by adding male *T*. *circumcincta*(*pinnata*/*trifurcata*) and female *Teladorsagia* spp. Female *Trichostrongylus* spp. nematodes were assigned based on location of recovery (i.e. abomasum or small intestine, respectively) to *T. axei* (abomasum) or *T. capricola*, *T. colubriformis* and *T. vitrinus* (small intestine). To estimate total counts per species for *T. capricola*, *T. colubriformis* and *T. vitrinus*, female *Trichostrongylus* spp. nematodes of the small intestine were proportioned according to the counts of males.

### Analysis of parasite and fecal egg counts: Studies 1, 2 and 3

For Studies 1 and 2, nematode counts by species and stage, if applicable, were transformed to the natural logarithm (ln) of (count +1) for calculation of geometric means for each treatment group. Efficacy was determined by calculating the percent efficacy as 100×[(C-T)/C], where C is the geometric mean nematode count among the untreated controls and T is the geometric mean among the animals treated with 0.5% *w*/*v* eprinomectin. The log counts for each nematode species of the treated group were compared to the log-counts of the control group using an F-test adjusted for the allocation blocks used to randomize the animals to the treatment groups. The mixed procedure in SAS version 9.4 was used for the analysis, with the treatment groups listed as a fixed effect, and the allocation blocks listed as a random effect. All testing was two-sided at the significance level α = 0.05.

For Study 3, fecal egg per gram (EPG) counts were transformed to the natural logarithm of (count + 1) for the calculation of geometric means by treatment group. Efficacy was determined based on post-treatment fecal egg counts by calculating the percent efficacy as 100×[(C-T)/C], where C is the geometric mean among the untreated controls and T is the geometric mean among the treated animals. The log-counts (EPG) of the treated group were compared to the log-counts of the untreated control group using analysis of variance for a generalized randomized block design. The mixed procedure in SAS version 9.4 was used for the analysis, with the treatment groups, sites and treatment-by-site interaction term listed as fixed effects and blocks as random effects. Exclusion criterion for individual analysis for nematodes was based on a rate of <40% animals shedding nematode eggs or lungworm larvae in the untreated controls. All testing was two-sided at the significance level α = 0.05.

### Collection and analysis of plasma and pharmacokinetic analysis: Study 4

In Study 4, whole blood of all sheep was collected from the jugular vein into lithium heparinized tubes prior to treatment (Day −1), and approximately 2, 4, 6, 8, 12, 24 and 36 h after treatment. Additional samples were collected on Days 2, 3, 4, 5, 6, 10, 14, 17 and 21. Plasma was separated by centrifugation and stored at ≤ − 20 °C until assayed for eprinomectin (B1a component) concentration.

All plasma samples collected were analyzed for eprinomectin B1a using a fully validated high-performance liquid chromatography method with fluorescence detection which was described previously [29]. The lower limit of quantitation of the assays for eprinomectin was established as 0.75 ng/mL, and the lower limit of detection of the assays as 0.50 ng/mL.

The analytical method performed well during sample analyses. Individual quality control (QC) samples had eprinomectin B1a recoveries in plasma from 89.0% to 110% for three QC levels: 1.0, 10 and 40 ng/mL; %relative standard deviation was 4.57 for 27 QC samples.

Pharmacokinetic analysis was performed using WinNonlin® version 5.2.1 non-compartmental analysis (Pharsight Corporation, Mountain View, CA, USA) for each individual animal and parameters were then averaged for the group. Eprinomectin plasma concentrations below the limit of quantitation of the assay method (<0.75 ng/mL) were not used in the pharmacokinetic calculations. The maximum concentration (C_max_) and time to maximum concentration (T_max_), and last quantifiable concentration (C_last_) and time to last quantifiable concentration (T_last_) were determined directly from the plasma concentration data. The first order rate constant associated with the terminal log-linear portion of the curve (k_el_) was estimated via linear regression of the log plasma concentration versus time curve and the terminal plasma half-life was calculated using T_1/2_ = ln(2)/k_el_. The area under the plasma concentration versus time curve (AUC) was determined using the linear trapezoidal rule for increasing plasma concentrations and the logarithmic trapezoidal rule for decreasing plasma concentrations (linear up/log down) from Day 0 to the last time the drug plasma concentration was above the lower limit of quantitation, AUC_last_. AUCs were also extrapolated to infinity using the formula: AUC_inf_ = AUC_last_ + C_last_/k_el_. The calculations were assessed by examining the extent of extrapolation for the AUC_inf_ values, so the AUC percentage extrapolated (AUC_%Extrap) was also determined. Group means and standard deviations were calculated.

## Results

No health problems or abnormal reactions to treatment were observed throughout the studies. In addition, all animals but one were reported to be healthy throughout the studies. This animal of Study 4 presented signs of respiratory disease at 18 and 19 days following treatment. It was thus medicated as appropriate and recovered within two days, and remained in the study until study end (Day 21).

### Studies 1 and 2 – nematode counts and efficacy

The nematode counts of 0.5% *w*/*v* eprinomectin-treated animals and the untreated control animals and percentage efficacy are summarized in Table 2 for those parasites which were recovered from at least four control animals in one of the two studies. For the sheep included in Study 2, pre-treatment fecal strongylid egg counts did not differ (*p* = 0.4725) between sheep allocated to the untreated control group and sheep allocated to the topical 0.5% *w*/*v* eprinomectin-treated group (range, 120 to 3010 EPG vs. 170 to 3130 EPG, respectively).

**Table 2:** Nematode counts and therapeutic efficacy against pulmonary and gastrointestinal nematodes of topical 0.5% *w*/*v* eprinomectin (EPRINEX® Pour-on, Merial) administered once at 1 mg/kg body weight to experimentally infected sheep (Study 1) or sheep with naturally acquired nematode infections (Study 2)

Study	Nematode counts				Probability^c^	Efficacy (%)^d^
	Control (untreated)		EPRINEX® Pour-on			
	NI/NG^a^	GM^b^ (Range)	NI/NG	GM (Range)		
*Dictyocaulus filaria*, adult						
1	10/10	30.1 (8–115)	0/10	0	<0.0001	100
*Chabertia ovina,* adult						
1	10/10	19.5 (5–70)	0/10	0	<0.0001	100
2	9/10	5.7 (0–38)	0/10	0	<0.0001	100
*Cooperia curticei*, adult						
1	10/10	99.4 (10–260)	0/10	0	<0.0001	100
2	9/10	674.9 (0–6820)	0/10	0	<0.0001	100
*Haemonchus contortus*, adult						
1	10/10	992.5 (450–1610)	0/10	0	<0.0001	100
2	9/10	477.2 (0–11,030)	0/10	0	<0.0001	100
*Nematodirus battus*, adult						
1	10/10	148.8 (10–450)	0/10	0	<0.0001	100
2	3/10	2.8 (0–130)	0/10	0	0.0465	100
*Oesophagostomum venulosum*, adult						
2	10/10	103.1 (3–893)	0/10	0	<0.0001	100
*Strongyloides papillosus, adult* ^*e*^						
1	10/10	151.4 (30–700)	0/10	0	<0.0001	100
*Teladorsagia circumcincta*(*pinnata/trifurcata*), adult						
1	10/10	1700.6 (950–3770)	0/10	0.0	<0.0001	100
2	10/10	6826.2 (1040–23,430)	3/10	1.7 (0–40)	<0.0001	>99.9
*Teladorsagia,* inhibited fourth-stage larvae						
1	3/10	1.0 (0–10)	0/10	0	0.0465	100
2	5/10	6.2 (0–240)	0/10	0	0.0214	100
*Trichostrongylus axei,* adult						
1	10/10	1169.3 (180–2430)	0/10	0	<0.0001	100
2	8/10	99.7 (0–1280)	0/10	0	0.0003	100
*Trichostrongylus colubriformis*, adult						
1	10/10	1032.3 (560–1400)	0/10	0	<0.0001	100
2	9/10	424.0 (0–13,947)	0/10	0	<0.0001	100
*Trichostrongylus vitrinus*, adult						
2	4/10	7.8 (0–146)	0/10	0	0.0155	100

^a^NI/NG: Number of sheep Infected/Number of sheep in Group

^b^GM = geometric mean, computed by subtracting 1 from the anti-logarithm of the mean of ln(count + 1)

^c^Probability using the F-Test

^d^Efficacy (%) = 100×[(GM Control – GM EPRINEX® Pour-on)/GM Control]

^e^Naturally acquired infection

Considering Studies 1 and 2 collectively, sheep treated with 0.5% *w*/*v* eprinomectin had significantly (*p* < 0.05) fewer of the following nematodes than the untreated control sheep with overall reduction of nematode counts by >99%: adult *D. filaria*, *H. contortus*, *T. circumcincta*(*pinnata/trifurcata*), *T. axei*, *T. colubriformis*, *Trichostrongylus vitrinus*, *C. curticei*, *N. battus*, *S. papillosus*, *Ch. ovina* and *O. venulosum*, and inhibited fourth-stage *Teladorsagia* larvae (Table 2).

Nematode parasites which were recovered from no more than three control animals per study and thus did not allow for a meaningful analysis were inhibited fourth-stage *Haemonchus* larvae (2/10 controls) in Study 1 and adult *Trichostrongylus capricola* (2/10 controls), *Capillaria musimon* (2/10 controls and 2/10 treated), *Trichuris ovis* (3/10 controls and 3/10 treated) and *Trichuris skrjabini* (3/10 controls and 1/10 treated) in Study 2. In addition, *Moniezia* cestodes were recovered from two controls and two 0.5% *w*/*v* eprinomectin-treated sheep in Study 2.

### Multicenter Field Study 3 – parasite counts of sentinel animals, fecal nematode egg counts and efficacy

All 196 sheep enrolled in the study at four sites were naturally infected with gastrointestinal nematodes. By pre-treatment fecal examination, strongylid, *Nematodirus*, *Trichuris* and protostrongylid nematode infections were demonstrated in 196, 37, 25 and 52 sheep, respectively. In addition, pre-treatment fecal examination revealed *Moniezia* cestode eggs in 31 sheep. Based on fecal examination, strongylid, *Nematodirus* and *Trichuris* nematode infections were present at all sites while evidence of protostrongylid lungworms and *Moniezia* cestodes was present only at Sites 2, 3 and 4 or Site 1, respectively.

Necropsy of two sentinel animals per site revealed a variety of gastrointestinal helminths (*H. contortus*, *T. circumcincta*(*pinnata/trifurcata*), *T. axei*, *T. capricola*, *T. colubriformis*, *T. vitrinus*, *N. battus*, *N. filicollis*, *Ch. ovina*, *O. venulosum*, *Tr. ovis*, *Tr. discolor* and/or *Moniezia* spp.) and/or *Protostrongylus rufescens* lungworms. The sentinel animals’ parasite counts, which defined the parasite composition of the study animals and represented the natural nematode contamination, indicated the occurrence of at least 12 and 11, four and six species of gastrointestinal nematodes at Sites 1, 2, 3 and 4, respectively (Table 3).

**Table 3:** Parasite counts of sentinel animals at Sites 1 to 4 of Study 3

Parasite species/stage	Parasite count							
	Site 1, Germany 1		Site 2, Germany 2		Site 3, Italy 1		Site 4, Italy 2	
	Animal 1	Animal 2	Animal 1	Animal 2	Animal 1	Animal 2	Animal 1	Animal 2
*Haemonchus contortus*, adult	60	40	20	1030	2890	760	160	20
*Teladorsagia circumcincta*(*pinnata/trifurcata*), adult	4860	3360	1660	8370	380	140	2460	70
*Trichostrongylus axei*, adult	40	150	350	1360	30	20	150	20
*Trichostrongylus capricola*, adult	25	0	0	107	0	0	120	60
*Trichostrongylus colubriformis*, adult	172	68	1193	4169	0	0	0	0
*Trichostrongylus vitrinus*, adult	123	23	47	214	50	40	20	720
*Nematodirus battus*, adult	270	290	0	10	0	0	0	0
*Nematodirus filicollis*, adult	100	290	0	20	0	0	0	0
*Moniezia* spp.	2	18	0	0	0	0	0	0
*Chabertia ovina*, adult	19	18	54	21	0	0	63	74
*Oesophagostomum venulosum*, adult	4	22	25	166	0	0	0	0
*Trichuris ovis*, adult	5	2	2	2	0	0	0	0
*Trichuris discolor*, adult	0	5	0	0	0	0	0	0
*Protostrongylus rufescens*, adult	0	0	0	229	0	0	0	0

Only strongylid egg counts were included in the analysis (Table 4). Analysis of strongylid egg counts did not reveal treatment-by-site interaction (pre-treatment, *p* = 0.9263; post-treatment, *p* = 0.0621); thus combined Sites 1 to 4 analysis of pre- and post-treatment strongylid egg counts comparing untreated control animals and topical 0.5% *w*/*v* eprinomectin-treated animals was performed. Pre-treatment fecal strongylid egg counts did not differ between the two groups (*p* = 0.2528). After treatment, topical 0.5% *w*/*v* eprinomectin-treated sheep had significantly (*p* < 0.0001) lower strongylid egg counts than the untreated control group across all sites. Reduction of strongylid egg counts was 98.6% across all sites and ≥97% at each site (Table 4). Pre-treatment coprocultures revealed larvae of the gastrointestinal nematode genera *Haemonchus*, *Teladorsagia* and *Trichostrongylus* for all sites while *Chabertia*/*Oesophagostomum* larvae were recovered from the coprocultures of Sites 1, 2 and 4 only. Identification of the larvae recovered from the post-treatment coprocultures of both untreated control animals and topical 0.5% *w*/*v* eprinomectin-treated animals at each study site indicated no change in the spectrum of nematode genera composition.

**Table 4:** Geometric mean fecal strongylid egg counts and percentage efficacy of topical 0.5% *w*/*v* eprinomectin (EPRINEX® Pour-on, Merial) administered once at 1 mg/kg body weight to naturally infected sheep under field conditions (Study 3)

Site(s)	Occasion	GM^a^ (Range) strongylid eggs per gram counts		Efficacy (%)^b^
		Control (untreated)	EPRINEX® Pour-on	
Site 1, Germany 1	Pre-Treatment^c^	437.7 (60–2150)	454.5 (70–1790)	NC^d^
	Post-Treatment^e^	276.2 (10–1490)	8.4 (0–120)	97.0
Site 2, Germany 2	Pre-Treatment	290.9 (90 – 3920)	416.0 (100–6480)	NC
	Post-Treatment	322.0 (90–3740)	5.7 (0–180)	98.2
Site 3, Italy 1	Pre-Treatment	1042.7 (108–4590)	1219.0 (126–12,456)	NC
	Post-Treatment	601.1 (162–6594)	12.1 (0–126)	98.0
Site 4, Italy 2	Pre-Treatment	633.0 (144–2064)	807.3 (480–4668)	NC
	Post-Treatment	519.9 (144–1200)	1.5 (0–36)	99.7
Sites 1 to 4 combined	Pre-Treatment	531.8 (60–4590)	650.8^f^ (70–6480)	NC
	Post-Treatment	406.4 (10–6594)	5.8^g^ (0–180)	98.6

^a^GM = geometric mean, computed by subtracting 1 from the anti-logarithm of the mean of ln(count + 1)

^b^Efficacy (%) = 100×[(GM Control – GM EPRINEX® Pour-on)/GM Control]

^c^Pre-treatment fecal examination, Day −5

^d^NC = Not calculated

^e^Post-treatment fecal examination, Day 14

^f^Control vs. EPRINEX® Pour-on, *p* = 0.2528

^g^Control vs. EPRINEX® Pour-on, *p* < 0.0001

*Nematodirus* eggs, *Trichuris* eggs and protostrongylid larvae were observed infrequently at fecal examinations with overall less than 40% of the animals in the control (untreated) group shedding eggs or larvae (Table 5) such that no meaningful analysis was possible.

**Table 5:** Fecal stages of intestinal and pulmonary nematodes in the naturally infected sheep of multicenter field Study 3 (Sites 1 to 4 combined) that were not analyzed because rate of detection was less than 40% in control (untreated) animals (*Nematodirus*, *Trichuris*, protostrongylid) and of *Moniezia* cestodes

Treatment group	Number of positive sheep/number of sheep in group							
	*Nematodirus* eggs		*Trichuris* eggs		Protostrongylid larvae		*Moniezia* eggs	
	PreT^a^	PostT^b^	PreT	PostT	PreT	PostT	PreT	PostT
Control (untreated)	11/49	8/49	4/49	8/49	12/49	14/49	6/49	4/49
EPRINEX® Pour-on	26/147	3/147	21/147	0/147	40/147	7/147	25/147	6/147

^a^PreT = pre-treatment fecal examination, Day −5

^b^PostT = post treatment fecal examination, Day 14

### Study 4 – pharmacokinetics of eprinomectin

**Fig. 1:**
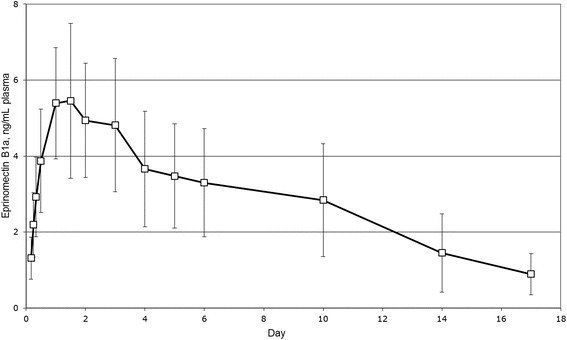
Plasma profile of eprinomectin B1a in sheep following topical administration of 0.5% *w*/*v* eprinomectin (EPRINEX® Pour-on, Merial) at 1 mg eprinomectin per kg body weight (Study 4). Each point represents the mean of plasma concentrations of eight sheep. Error bars indicate standard deviations

The absence of eprinomectin (B1a component) was confirmed in the plasma samples of the animals prior to treatment with topical 0.5% *w*/*v* eprinomectin. The plasma concentration vs. time profile of eprinomectin following treatment is shown in Fig. 1, and the pharmacokinetic parameters are summarized in Table 6. Eprinomectin B1a was detected in the plasma of all sheep at quantifiable levels four hours after treatment and remained at quantifiable levels in all animals until Day 10 when the average concentration was 2.84 ± 1.48 ng/mL. The highest mean plasma eprinomectin (B1a component) level (5.46 ± 2.04 ng/mL) was observed 36 h post treatment followed by a continuous decline until Day 21 when three animals had quantifiable levels (0.804–1.03 ng/mL). Greater than 20% extrapolation (AUC_%Extrap) of the total AUC in four sheep indicates that the elimination phase was not adequately defined in these animals. Based on the four animals in which the elimination phase was adequately defined, AUC_inf_ was 69.8 ± 13.7 day*ng/mL.

**Table 6:** Basic pharmacokinetic parameters describing the disposition of eprinomectin (B1a component) in plasma of sheep after administration of topical 0.5% *w*/*v* eprinomectin (EPRINEX® Pour-on, Merial), current Study 4 and data from other authors

Source	Topical eprinomectin (mg/kg body weight)	C_max_ (ng/mL)	T_max_ (day)	T_1/2_ (days)	AUC_last_ (day*ng/mL)
Current Study 4	1.0 (*n* = 8^a^)	6.20 (± 1.71)	3.13 (± 2.99)	6.40 (± 2.95)	48.8 (± 19.2)
[19]	0.5 (*n* = 6^b^)	2.22 (± 0.88)	1.2 (± 0.4)	5.4 (± 0.7)	13.6 (± 4.8)
	1.0 (*n* = 6^c^)	5.25 (± 2.7)	1.5 (± 0.5)	12.2 (± 5.8)	33.7 (± 22.5)
[16]	0.5 (*n* = 6^d^)	NR^e^	NR	NR	56.0 (± 26.2)
[18]	0.5 (n = 6^f^)	2.28 (± 0.41)	3.17 (± 0.40)	2.20 (± 0.34)	16.2 (± 3.69)
	0.5 (n = 6^g^)	2.30 (± 0.60)	3.00 (± 0.45)	1.85 (± 0.13)	15.5 (± 3.67)

^a^Female dry adult Merino Cross sheep, sampled up to 21 days post dose

^b^Female lactating adult Istrian Parmenka sheep, sampled up to 32 days post dose

^c^Female lactating adult Istrian Parmenka sheep, sampled up to 42 days post dose

^d^Five month old sheep, sampled up to 21 days post dose

^e^Not reported

^f^Female lactating (early-mid lactation) adult Pampina Cross sheep, sampled up to 35 days post dose

^g^Female lactating (mid-late lactation) adult Pampina Cross sheep, sampled up to 35 days post dose

## Discussion

The primary objective of the studies was to confirm the efficacy of the 0.5% *w*/*v* eprinomectin formulation (EPRINEX® Pour-on, Merial) against gastrointestinal and pulmonary nematode endoparasites in sheep when administered at 1 mg eprinomectin per kg body weight. Based on parasite burdens recovered from the sheep with induced and naturally acquired nematode infections and the reduction of fecal egg counts in the multicenter field study including the parasite counts of sentinel animals from all sites, results of this series of studies demonstrated consistently a very high efficacy against the major production-limiting gastrointestinal nematode parasites affecting sheep in temperate climates, i. e. *H. contortus*, *T. circumcincta*(*pinnata/trifurcata*), *T. axei*, *T. colubriformis*, *T. vitrinus*, *C. curticei*, *N. battus*, *Ch. ovina* and *O. venulosum* [8, 11], and *D. filaria* lungworms. These species of nematodes are representative of the spectrum of nematode parasites infecting sheep throughout Europe and are found to a greater or lesser extent in sheep in southern Europe, e. g. Spain, Italy and Greece [15, 30–32], and central and northern Europe, e. g. Austria, Czech Republic, Germany, the UK and Norway [33–38]. Parasitism of naturally infected sheep determined in the context of the studies reported here demonstrates that gastrointestinal nematode infections remain an important constraint to sheep in Europe such that appropriate control measures including anthelmintic use are needed to ensure appropriate levels of productivity as well as animal welfare [10, 11]. As shown with respect to dairy cattle, the availability of a broad spectrum anthelmintic for use in sheep (and goats) with a zero hours milk withholding period offers an unique advantage for the treatment of lactating animals which have been demonstrated to benefit substantially from efficacious nematode control [2, 4–7].

The results of the dose confirmation laboratory studies and the multicenter field study indicate some variability in efficacy in that, compared to untreated animals, sheep treated with topical 0.5% *w*/*v* eprinomectin demonstrated >99% efficacy with respect to nematode count reductions while efficacy in terms of reduction of fecal egg counts varied from 97% to >99% at the field study sites. Considering that fecal cultures suggested no change in the spectrum of nematode population composition following treatment at the field study sites, this finding may, at least partly, reflect variability in the sensitivity of the respective nematode populations.

The high efficacy against all major gastrointestinal and pulmonary nematodes of sheep demonstrated in the present studies adds considerable knowledge regarding the spectrum of nematocidal activity of topical 0.5% *w*/*v* eprinomectin compared to observations reported previously which yielded three nematode species from one necropsy study [16] and gastrointestinal nematode egg and lungworm larval count reductions from field efficacy evaluations [15, 17, 20]. In addition, there is also indication of efficacy of topical 0.5% *w*/*v* eprinomectin against *Oestrus ovis* nasal bot infestation [16, 17]. Overall, the therapeutic efficacy demonstrated in the present studies in sheep was very similar to the array of nematode parasites effectively treated by the administration of topical 0.5% *w*/*v* eprinomectin at 1 mg per kg body weight to goats [39, 40].

Any anthelmintic use raises concerns in terms of selection of resistant parasite populations. Recently published systematic reviews of peer-reviewed literature concluded that anthelmintic resistance in gastrointestinal nematodes of sheep is generally widespread in Europe but prevalence varies importantly by region and class of anthelmintic [41] and that high frequency of treatments is the major risk factor associated with anthelmintic resistance in sheep [42]. Therefore, monitoring the efficacy of treatments, appropriate grazing management, and exclusion of part of the nematode population from the exposure to the treatment (creation and/or maintaining of refugia) may be ways to reduce the selective advantage for resistant specimens. Overall, sustainable control requires responsible use of correctly administered anthelmintics providing a balance between maintaining acceptable levels of productivity and animal welfare and the inevitable evolution of anthelmintic resistance because the risk of losses from parasite infection may increase with further intensification of pastoral production systems [11, 42–44].

Regarding induced infection Study 1, inoculation produced adequate levels of infections as recommended by VICH GLs 7 and 13 [22] for all nematodes but *O. venulosum* which was not recovered from any animal. This finding is probably related to an antagonistic interaction between *Ch. ovina* and *O. venulosum* which is dominated by *Ch. ovina*. Both species of large intestinal nematodes under natural infection conditions frequently occur in co-infections [33, 34, 36, 45; this Study 2]. However, infection with *Ch. ovina* stimulates an immune response in the host and suppression of *O. venulosum*, when inoculated subsequently to challenge with *Ch. ovina,* has been observed previously in experimental studies [46, 47]. In addition to the nematodes inoculated, all untreated control animals harboured *Strongyloides papillosus* nematodes. As fecal samples of the lambs were negative for *Strongyloides* eggs before initiation of experimental infections, this inadvertent infection originated likely either from pre-patent infections present in at least some lambs at the time of the pre-inoculation fecal examination or lambs harbored very low level patent infections resulting in egg excretion below the detection limit of the McMaster method used for examination of the feces. *Strongyloides papillosus* is transmitted through the bedding (eggs can hatch in the bedding and third-stage larvae infect sheep by skin penetration) [48] such that infection may have spread among the study animals during the seven week indoor-housing period prior to treatment.

Plasma concentrations and basic pharmacokinetic parameters were comparable to those previously reported following the administration of topical 0.5% *w*/*v* eprinomectin at 1 mg per kg body weight to sheep [19]. Although some variability can be seen possibly due to different animal physiology (e.g., lactating vs. non-lactating) or breed, considering data of adult female sheep treated with topical 0.5% *w*/*v* eprinomectin at 0.5 mg per kg body weight [18, 19] indicates dose proportionality. However, one study indicated an exceptional high AUC_last_ of 56.0 ± 26.2 day*ng/mL following topical administration of 0.5% *w*/*v* eprinomectin at 0.5 mg/kg body weight to five months old lambs weighing 20 to 25 kg [16]. Results of this study are difficult to interpret as only limited information on the pharmacokinetic profile and characteristics of study animals was reported. Compared to goats [cf. 40], T_max_ appears to occur later in sheep, indicating slower absorption possibly due to the difference of the structure of the skin/hair coat characteristics between the two species. Overall, similar pharmacokinetic profiles were demonstrated for topical 0.5% *w*/*v* eprinomectin in sheep and goats which translate to a similar spectrum of anthelmintic activity in sheep (these Studies 1, 2 and 3) and goats [39, 40, 49].

## Conclusion

This series of studies demonstrated eprinomectin administered topically at 1 mg/kg body weight onto the skin of sheep to be highly efficacious against a broad range of ovine gastrointestinal nematodes and *D. filaria* lungworms and to be well tolerated by sheep of different ages, breeds, gender and physiological status.
